# Tuning the Solvation and Solubility Properties of Molecularly Heterogeneous Nonionic Deep Eutectic Solvents via Interface Organization

**DOI:** 10.1021/acs.langmuir.5c05846

**Published:** 2026-03-20

**Authors:** Laura X. Sepulveda-Montaño, Chaila N. Estrella, Amelia M. Skinner, Daniel G. Kuroda

**Affiliations:** Department of Chemistry, 5779 Louisiana State University, Baton Rouge, Louisiana 70803, United States

## Abstract

Common separation techniques, such as liquid–liquid extraction, are usually used for extractions and purifications due to their industrial scalability and affordability. However, these well-established practices are hindered by low selectivity and challenges in recovering solutes and solvents. Deep eutectic solvents (DES), a fairly new type of solvent, have the potential to overcome these issues. DESs are binary mixtures whose physical properties can be tuned by selecting the appropriate precursors to facilitate and/or enhance processes such as extraction. A promising DES for selective separations is formed when lauric acid (LA) is mixed with *N*-methylacetamide (NMA). This LA-NMA DES has a heterogeneous microscopic structure that can solvate compounds with completely different polarities. This study explores how forming an organized structure on the mesoscale affects the solubility of nonpolar solutes in nonionic DESs. To this end, the molecular and mesoscale structures and their effect on the solubility and solvation properties are evaluated for the LA-NMA DES and two new DESs with slight chemical variations in their precursors. It is observed that the organization of the nonpolar DES domains and, consequently, of their interfaces directly relates to the solubility of nonpolar compounds. Specifically, correctly selecting the DES precursors that form organized nonpolar domains leads to an organized interface in which the nonpolar solutes are solvated, thereby increasing the solubility. Additionally, enhanced dissolution power was observed in a completely different DES with mesoscale order in its molecular structure and composed of menthol and lauric acid. The latter result further validates the proposed tunability of the DES dissolution power through organized interfaces, extending it beyond a specific DES family and opening the possibility of new extraction-tailored designer solvents.

## Graphical abstract

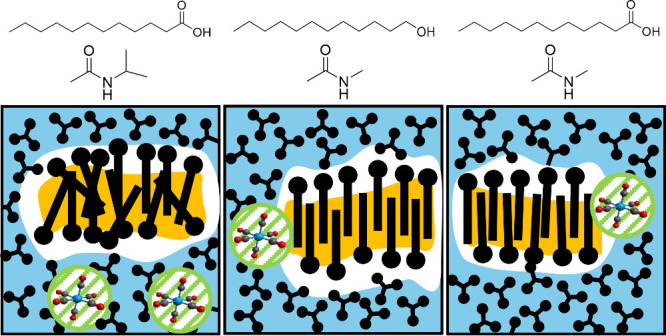

## Introduction

The extraction and separation of chemical compounds is a key step in numerous industrial processes.^1–4^ The separation process is typically challenging due to various factors, such as the chemical nature of both the compounds^5^ and the matrix containing them,^6–9^ the cost of manufacturing the materials used for the separation,^10^ etc.^11–13^ Hence, many new separation and extraction methods have been developed recently,^14^ but absorption and/or solubilization remain widely used due to their simplicity.^15,16^ To this end, improving the capabilities of the materials used for the extraction and separation of chemical compounds has remained of utmost importance.^4,17–19^

The separation of solutes from matrices via absorption usually requires manipulating the shape, porosity, and chemical nature of the material surface to enhance its selectivity.^20–23^ However, recovering and recycling these materials is very challenging.^24,25^ In contrast, solvent extraction is comparatively simple, as it only relies on the ability of the solvent to interact with the matrix. This ability usually depends on the solvent density,^26,27^ viscosity,^28–30^ chemical nature,^31^ etc.^32,33^ Nevertheless, using common extraction solvents is generally expensive, since most require proper disposal. To address these issues, many recent efforts have focused on using designer solvents, which can be easily discarded and/or recycled.^34^

In the last few decades, new designer solvents with increased separation efficiency have emerged. Examples include ionic liquids and deep eutectic solvents (DES). These designer solvents can be tailored for different applications by carefully selecting their molecular composition.^35–39^ However, the low cost of DES production and the possibility of making them from natural feedstocks give DESs an advantage over ionic liquids.^40,41^ DES synthesis usually involves simply mixing two high melting point compounds as their mixtures form a liquid at room temperature, but are characterized by a single-phase transition endotherm in agreement with the presence of a eutectic point.^42^ At the so-called eutectic composition, these mixtures experience the most significant decrease in melting temperature, accompanied by a melting curve similar to that of a single-component system.^43^ Beyond the DES liquid state at room temperature, the DESs are versatile because of the wide variety of precursor combinations,^35,41,44^ which provides vast opportunities for finding mixtures with the appropriate physical properties, such as low density, viscosity, high conductivity, etc.^42–45^ Moreover, the binary composition of DESs provides an additional degree of freedom that can be altered to specifically tailor the properties of the mixture for a given application, such as extraction.

DESs have shown potential in extraction and separation processes. For example, DESs can efficiently separate oil from water with an efficiency comparable to state-of-the-art gelators usually used in oil spill remediation.^46^ An example of these promising DESs is the one formed by mixing lauric acid (LA) and *N*-methylacetamide (NMA) (Figure 1).^46^ The LA-NMA mixture has a eutectic composition of a 1:4 molar ratio of LA to NMA, with a melting temperature of ∼6 °C. Additionally, to this eutectic composition, the LA-NMA mixtures with molar ratios between 1:2 and 1:6 are also liquids at room temperature.^47^ When an LA-NMA DES interacts with an oil–water mixture, the NMA dissolves in the water, which triggers the precipitation of LA and generates the nonpolar matrix necessary for the oil to form a gel. This gel can be easily separated from the aqueous phase by simple decantation or filtration.^46^ This mechanism is vital not only for the separation properties of the LA-NMA DES but also for the recovery of the precursors, as the individual components can be separated by the addition of water.

**Figure 1:**
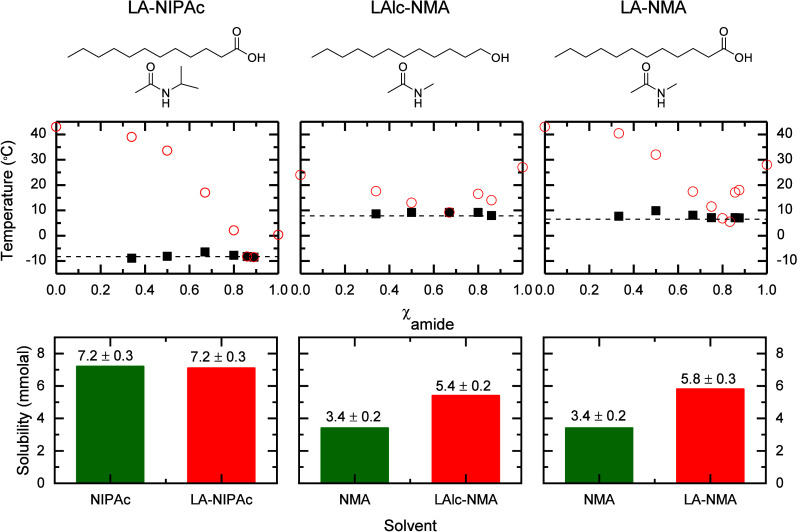
From top to bottom: First row contains the molecular structures of DES precursors, second row contains the phase diagrams of their mixtures at various molar ratios, and third row contains the solubility of W­(CO)_6_ in each DES (red) and its amide precursor (green) at 35 °C. Each column showcases the data for the DES formed by chemical compounds on the first row (i.e., LA-NIPAc (left), LAlc-NMA (center), and LA-NMA (right)). In the phase diagrams, black squares and red circles represent the first and second transition temperatures of each mixture; also dashed lines represent the approximate eutectic melting temperature. The phase diagram of LA-NMA is shown for comparison purposes only. Reproduced from ref 47. Copyright 2019 American Chemical Society.

Characterization of the molecular structure by small-angle X-ray scattering (SAXS) has revealed that the LA-NMA DES is not an ideal solution since it has a nanoheterogeneous mesoscale structure.^47^ In particular, SAXS characterization has shown that the DES consists of LA nonpolar domains in a polar matrix of NMA.^48^ Later studies demonstrated that the LA domains are stabilized by hydrogen bonds between precursors at the interface and by the formation of stable LA crystalline-like domains where the acid carboxylic alkyl chains interact in an interdigitated arrangement.^47,49,50^ These findings have been critical to explain the ability of the LA-NMA DES to separate and extract compounds based on polarity.^51^ Interestingly, these studies also showed that the solvation of medium- to low-polarity compounds occurs at the interface between LA and NMA domains rather than within the nonpolar LA domain.^49–51^ These unexpected results are a consequence of the high organization of the LA aggregates, which does not allow the insertion of nonpolar molecules into the LA (nonpolar) region.^49–51^

The interaction of the different solutes with the nonpolar–polar interface in DESs is complex. Molecular dynamics simulations have shown the existence of not only the domain boundaries or interfaces but also channels within the LA domain where the different solutes can be located. This expands the range of accessible interfacial environments for solutes.^49,50^ Overall, the solvation studies of different compounds demonstrated that the interface between polar (NMA) and nonpolar (LA) domains is crucial for the LA-NMA DES to accommodate nonpolar solutes within its heterogeneous molecular structure.

The formation of nanoscopic heterogeneities and the subsequent interface formation between DES precursors is a well-known phenomenon.^48,52–60^ While the use of heterogeneous DESs for solubilization and extraction of compounds has been extensively studied,^55,61–66^ the impact of the formation of interfaces in nanoheterogeneous DESs, as well as the manipulation of their structural organization and its effect on the solvation properties of solutes, is, to the best of our knowledge, yet to be demonstrated. This report examines how the interface between polar and nonpolar domains in molecularly heterogeneous nonionic DESs affects the solvation of nonpolar solutes and modulates their solubility capabilities. This tuning of the DES solubility is achieved by making structural changes to the original DES components, namely, NMA and LA. Specifically, two modifications to the original precursors are evaluated (Figure 1). The first modification replaces the amide component, NMA, with *N*-isopropylacetamide (NIPAc). The isopropyl group of the amide disrupts the hydrogen bond network in liquids,^67^ and so, it is expected to destabilize the polar domains of the DES, along with the nonpolar domains and their interface. The second modification slightly alters the nonpolar component (LA) by replacing its carboxylic acid (headgroup) with an alcohol (lauryl alcohol or LAlc). The latter chemical modification is expected to affect the hydrogen bond capacity of the nonpolar precursor but not the formation of domains, or equivalent, molecular heterogeneities.

## Experimental Section

### Sample Preparation

DESs were prepared using lauric acid (Alfa Aesar, 99.5%), *N*-methylacetamide (Alfa Aesar, 99%), *N*-isopropyl acetamide (Ambeed, 99.98%), lauryl alcohol (Sigma, ∼99%), menthol (Beantown Chemical, 99%), benzyl thiocyanate (TCI, 95%), tungsten hexacarbonyl (Pressure Chemical Co, 98%), and octanoic acid (Acros Organics, 99%). These chemicals were used as received without further purification. DES components were mixed at different molar ratio compositions, as described in the text, in glass vials with a vortex until a clear liquid formed.

The samples for the determination of solubility were prepared by adding W­(CO)_6_ to each DES mixture until an excess of solid was observed. Then, the samples were heated at 35 °C overnight, followed by filtration using 0.22 μm PTFE filters.

All samples were prepared and stored in a nitrogen-fluxed glovebox. For details in the determination of the solubility of W­(CO)_6_, please see the Sample Preparation section of the Supporting Information, Table S1, and Figure S1.

### Differential Scanning Calorimetry (DSC)

The DSC measurements were performed on a DSC 250 (TA Instruments), using an empty pan as a reference. The sample (∼0.5 mg) and the reference were held in alodined aluminum pans (TA Instruments). The DSC thermograms were obtained by cool-heat–cool-heat cycles from −20 to 40 °C for the LAlc-NMA DES and −80 to 60 °C for the LA-NIPAc DES, both at a rate of 2 °C/min.

### Small-Angle X-ray Scattering (SAXS)

SAXS measurements were performed by using a Cu Kα laboratory X-ray source (Genix from Xenocs) with a wavelength of 1.54 Å at the Louisiana State University center for advanced microstructures and devises (CAMD). Samples were loaded into 1 mm diameter borosilicate capillary tubes for all measurements with a sample to detector distance of 263.4 mm.

### Fourier Transform IR (FTIR) Spectroscopy

The FTIR measurements were performed in a Bruker Tensor 27 with a liquid nitrogen cooled MCT detector and 0.5 cm^–1^ resolution. The spectra are the results of averaging over 40 scans. The samples were held in an O-ring sealed cell between two CaF_2_ windows separated by Teflon spacers with thickness such that the absorbance was less than 1.0 OD. The solubility samples were measured at 35 °C in a homemade temperature-controlled cell.

## Results and Discussion

Binary mixtures of both LAlc-NMA and LA-NIPAc present depressions in their melting points with respect to the melting point of the precursors (43, 24, 28, and 0 °C for LA, LAlc, NMA, and NIPAc, respectively), as demonstrated by their phase diagrams (Figure 1) obtained through DSC (see Figure S2). In particular, these mixtures have eutectic points with a 1:2 molar ratio (33.3% of organic alcohol) for LAlc-NMA and a 1:6 molar ratio (14% of organic acid) for LA-NIPAc, which differ from the 1:4 molar ratio (20.0% of organic acid) of the original LA-NMA DES. Overall, these findings show that the chemical modifications to the original DES precursors do not inhibit the formation of deep eutectics.

The ability of the LAlc-NMA and LA-NIPAc DESs to solvate and, consequently, to extract nonpolar compounds was evaluated by determining the solubility of the nonpolar solute (tungsten hexacarbonyl, W­(CO)_6_) using FTIR spectroscopy. The results shown in Figure 1 and Table S1 show that the solubility of W­(CO)­6 in the NMA-based DESs is greater than that in pure NMA, with an increase in solubility of more than 36% in both NMA-based DESs with respect to the pure amide. In contrast, the solubility of the nonpolar compound is reduced by less than 1% in the LA-NIPAc DES when compared to that of pure NIPAc (Figure 1 and Table S1). Finally, the solubility of the nonpolar compound in the LA-NMA DESs increases with the LA concentration (Table S1 and Figure S3). The solubility experiments appear to show two different results for the LAlc-NMA and LA-NIPAc DESs. First, the presence of long alkyl chain components increases the solvation capabilities of DESs containing NMA for nonpolar solutes. Second, the presence of LA in the LA-NIPAc DES does not appear to confer any special dissolution properties with respect to the pure amide precursor. It is important to note that the nonpolar solute is more soluble in NIPAc than in the other mixtures. Hence, this DES precursor does not require the formation of a DES, which is often necessary since most DES precursors are solid at room temperature.

The contrasting solubility of a nonpolar solute among DESs must arise from the differences in their structure. As previously mentioned, the LA-NMA DES has nonpolar domains stabilized by hydrogen bond interactions occurring primarily at the LA-NMA interface, where the nonpolar solutes are located.^47^ The replacement of NMA with NIPAc is likely to destabilize the amide domain since NIPAc is a hydrogen bond disruptor.^67^ A similar case can be made for replacing LA with LAlc since the alcohol has reduced hydrogen bond capabilities when compared to LA. Consequently, it is possible that the destabilization of the polar and/or nonpolar domains affects the overall structure of the resulting DES (Figure 2) and thus its solvation properties. To evaluate this possibility, the interaction between moieties of the DES precursors was investigated using FTIR spectroscopy (Figure 2). Three different compositions (1:2, 1:4, and 1:6 LA to NMA molar ratios) were chosen as their comparison allows us to identify changes in the chemical environment as a function of the molar ratio of components.^47^ In particular, the amide and the carbonyl groups have vibrational modes, such as amide I and the carbonyl stretch, located in the 1500 to 1800 cm^–1^ region, which serve as molecular reporters of hydrogen bond interactions, as previously demonstrated.^67–72^ Surprisingly, the FTIR spectrum of the LA-NIPAc mixtures is very similar to that of the LA-NMA mixtures (Figure 2 and Figure S4), albeit with the difference in the amide I band of the amides due to their excitonic nature.^72^ This result indicates that most of the intermolecular interactions, such as hydrogen bonds, occurring between LA and NMA are conserved in the LA-NIPAc DES. This statement is supported by the temperature-dependent FTIR for this sample (Figure S5), which closely resembles the results for the LA-NMA DES.^47^ These IR spectra in the amide I region show an increase and decrease of intensity at low and high frequencies, respectively, due to the formation of more hydrogen bonds at low temperature.^47^ The largest change in the IR spectra of both the LA-NIPAc and LA-NMA DESs is associated with the rise of the low frequency band in the amide I region (∼1620 cm^–1^), due to the formation of hydrogen bonds at the LA-NIPAc interface (see interaction map in Figure 2). In contrast, the spectrum of LAlc-NMA DESs presents only minor changes compared to the pure amide precursor. Despite the modest changes in the IR spectral features when transitioning from the pure NMA to the LAlc-NMA DES, some spectral features indicate that the amide group perceives the presence of the alcohol. For example, the rise in the amide I band intensity in the LAlc-NMA DES denotes a lack of interactions between the amide precursor and the LAlc headgroup (Figure 2), which results in more free NH groups at the nonpolar–polar interface compared to the LA-NMA DES. The latter result is consistent with LAlc containing only an alcohol group as a hydrogen bond donor/acceptor, whereas LA has both a carbonyl and a hydroxyl group that can serve as hydrogen bond donors/acceptors and interact with the amide molecules (see interaction map in Figure 2). Overall, the FTIR spectra of the LAlc-NMA and LA-NIPAc DESs expose differences related to the formation of nonpolar domains, or equivalent, hydrogen bonds at the interface. Therefore, the change in the solubility of nonpolar solutes in the LAlc-NMA and LA-NIPAc DESs is likely not caused by any significant changes in the intermolecular interaction potential of the DES precursors but rather by the differences in the nonpolar domains.

**Figure 2:**
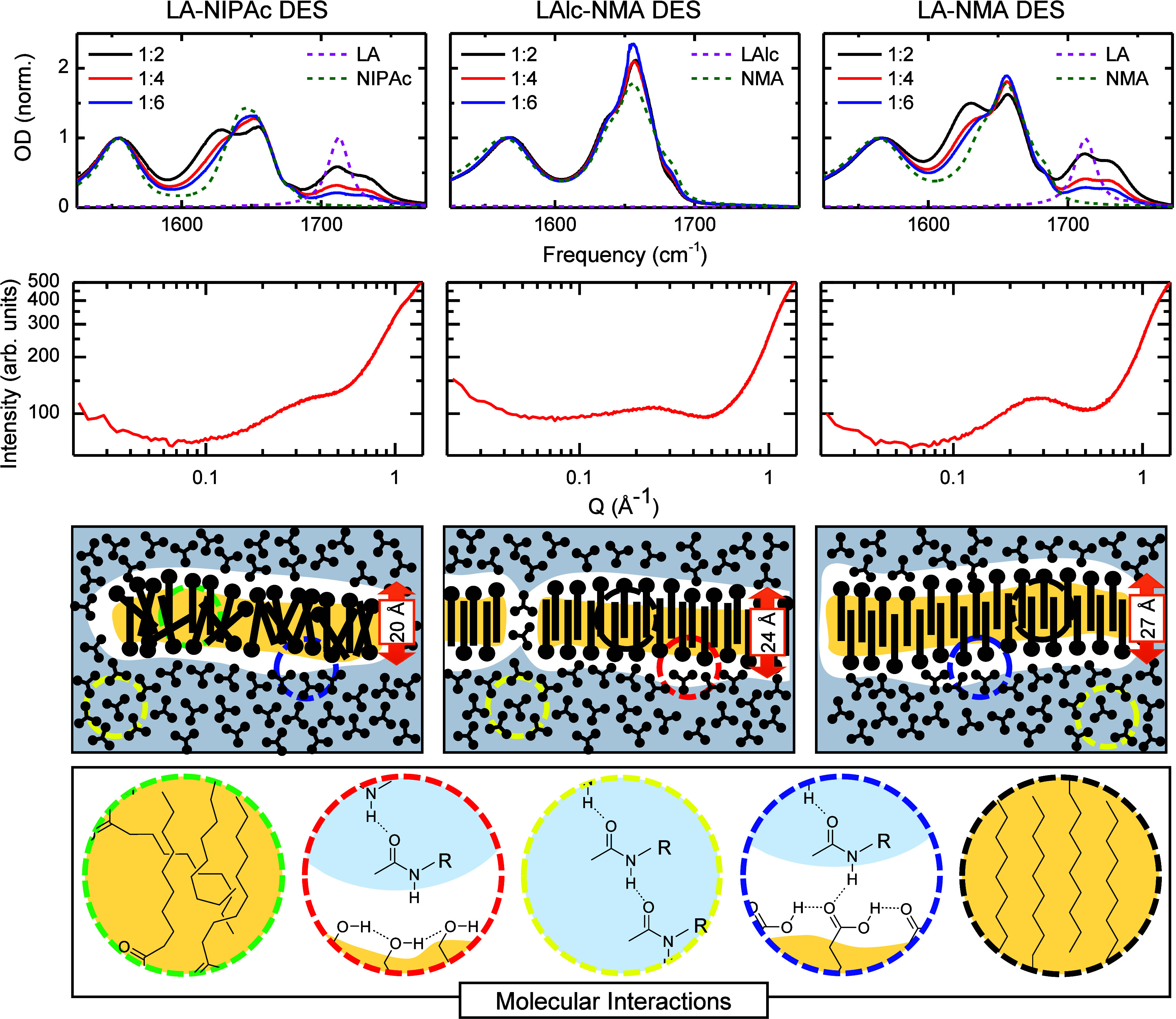
Molecular and mesoscopic characterizations of the modified organic acid-amide DES. The three columns correspond to LA-NIPAc (left), LAlc-NMA (center), and LA-NMA (right). First row shows the FTIR spectra normalized with respect to the amide II mode, the lines represent the mixtures in the 1:2 (black), 1:4 (red), 1:6 (blue) molar ratios, pure amides (NMA, and NIPAc in green), and pure organic acids (LA, and LAlc in pink). Second row shows the SAXS profiles of various eutectic mixtures at 1:4 molar ratios. The third row shows a cartoon representation of the mesoscopic structure of the DESs, the polar domains are blue, nonpolar are yellow, and their interface is white). Fourth row shows the possible molecular interactions responsible for the experimental signals (in the same colors as in the third row). The LA-NMA DES FTIR shown in first row is for comparison purposes only. Reproduced from ref 47. Copyright 2019 American Chemical Society.

FTIR spectroscopy provides information about short-range intermolecular interactions. However, it does not describe the mesoscale ordering of the system, which has been shown to play a major role in the dissolution of nonpolar and low-polarity solutes.^49–51^ Hence, the mesoscale structures of the DESs were evaluated by using X-ray scattering (i.e., SAXS). The SAXS profiles of the three DESs at a 1:4 composition (Figure 2) contain a prepeak between 0.1 and 0.6 Å^–1^, which is a signature of nanoscale heterogeneities.^49^ This molar ratio was chosen for structural characteristics since the LA-NMA DES is very well-characterized at this composition.^47,49–51^ This allows one to observe the structural changes in heterogeneities of the LAlc-NMA and LA-NIPAc DESs with respect to the LA-NMA system driven exclusively by the chemical modifications of the precursors. These prepeaks are attributed to domains formed by the aggregation of the nonpolar precursor, either LA or LAlc. Moreover, the low Q scattering peak matches the distances previously measured for the separation of polar heads in n-alcohols of different lengths,^73–75^ as well as in the original LA-NMA DES, indicating that the polar heads of the nonpolar precursors give rise to this prepeak.^49^ Consequently, the results demonstrate that the LAlc-NMA and LA-NIPAc DESs exhibit mesoscale heterogeneities in their structure similar to those of the LA-NMA DES, due to the aggregation of the nonpolar DES precursor. These scattering profiles (Figure 2) also reveal changes in the size of the aggregates, as seen by the variation in the position of the prepeak maxima (i.e., *Q*_max_ value). Specifically, the prepeak in the LAlc-NMA and LA-NIPAc DESs indicates that the domains of LA-NIPAc and LAlc-NMA have smaller (*Q* = 0.31 Å^–1^ or equivalent 20.3 Å in real space) and larger (*Q* = 0.23 Å^–1^ or equivalent 27.3 Å in real space) distances between polar heads of LA and LAlc, respectively. Interestingly, the smaller distance between polar heads in the LA-NIPAc DES compared to the LA-NMA DES suggests volume contraction and a potential loss of the highly organized LA structure observed in the latter (Figure 2). In contrast, the LAlc-NMA DES appears to have a slightly enlarged nonpolar domain.^49^ The intensity of the SAXS prepeak validates the proposed level of organization of the nanoscopic domains in the LA-NIPAc and LA-NMA DESs since more organized domains produce larger scattering signals.^76,77^ Hence, the SAXS profiles show that the LA-NIPAc nanodomains are more disorganized than those of the LA-NMA DES as seen by the height of their prepeaks (Figure 2). While the comparison between prepeak intensities is possible in the LA-NMA and LA-NIPAc DES because they only differ by two methyl groups in NIPAc (Figure 1) that do not contribute to the scattering intensity of the prepeak,^75^ the difference in the head groups between LA and LAlc, and consequently, in their X-ray cross sections, forbids any direct comparison of the prepeak intensity of their corresponding DESs. Overall, the mesoscale characterization shows that the LA-NIPAc DES likely lost the alkyl tail ordering in the nonpolar domains (i.e., they are more disorganized), while LAlc-NMA still maintains a similar mesoscopic ordering.

The changes in the molecular organization of the nonpolar domains in the three DESs play a critical role in the solubility of nonpolar solutes because nonpolar molecules are solvated in the interface between domains.^49,50^ The LA-NIPAc DES presents a slight decrease in solubility of nonpolar compounds when compared to the pure NIPAc, while the LAlc-NMA DES exhibits an enhanced solubility as compared to the original LA-NMA DES. Therefore, it is concluded that the change, or lack thereof, in the solubility of nonpolar compounds in the LAlc-NMA and LA-NIPAc DESs, compared to their pure polar precursors, is due to the presence, or absence, of a highly organized interface between the polar and nonpolar domains.

The presence of the organized interface and its impact on the solubility of the nonpolar solutes in the DESs were further validated by assessing the location of the solutes within their molecularly heterogeneous structures. To this end, FTIR spectroscopy provides a reliable and straightforward tool for assessing the chemical/solvation environment of molecules.^51^ In particular, the FTIR spectra of different solutes in LA-NMA DESs showed that when the molecular environment of a compound does not change with solvent composition, its IR bands remain mostly unaffected.^51^ Therefore, FTIR spectroscopy was used to investigate the solvation environment of the polar and nonionic solute benzyl thiocyanate (BSCN) as well as the nonpolar solute (W­(CO)_6_) in the LAlc-NMA and LA-NIPAc DESs. These compounds are known to be located at the nonpolar/polar interface in the original LA-NMA DES.^51^ The results for both BSCN and W­(CO)_6_ in the LA-NIPAc DESs (Figure 3) show that the nitrile and carbonyl stretches, respectively, remain remarkably invariant to the LA concentration, as demonstrated by the lack of changes in both the central frequency and bandwidth (see Tables S2 and S3 and Figure S6). In contrast, the spectral features of both compounds clearly evolve in the LAlc-NMA mixtures. For BSCN, a blueshift and a broadening of the nitrile stretch band occur with the addition of LAlc (see Tables S2 and S3, Figure S6, and Figure 3). The nonpolar W­(CO)_6_ in LAlc-NMA mixtures also shows a similar IR spectral evolution pattern as a function of the nonpolar component concentration as BSCN does (Figure 3).^51^ Most remarkably, the IR bands of both solutes in the LAlc-NMA mixtures closely resemble those in the original LA-NMA DES (Figure 3 and Tables S2 and S3).

**Figure 3:**
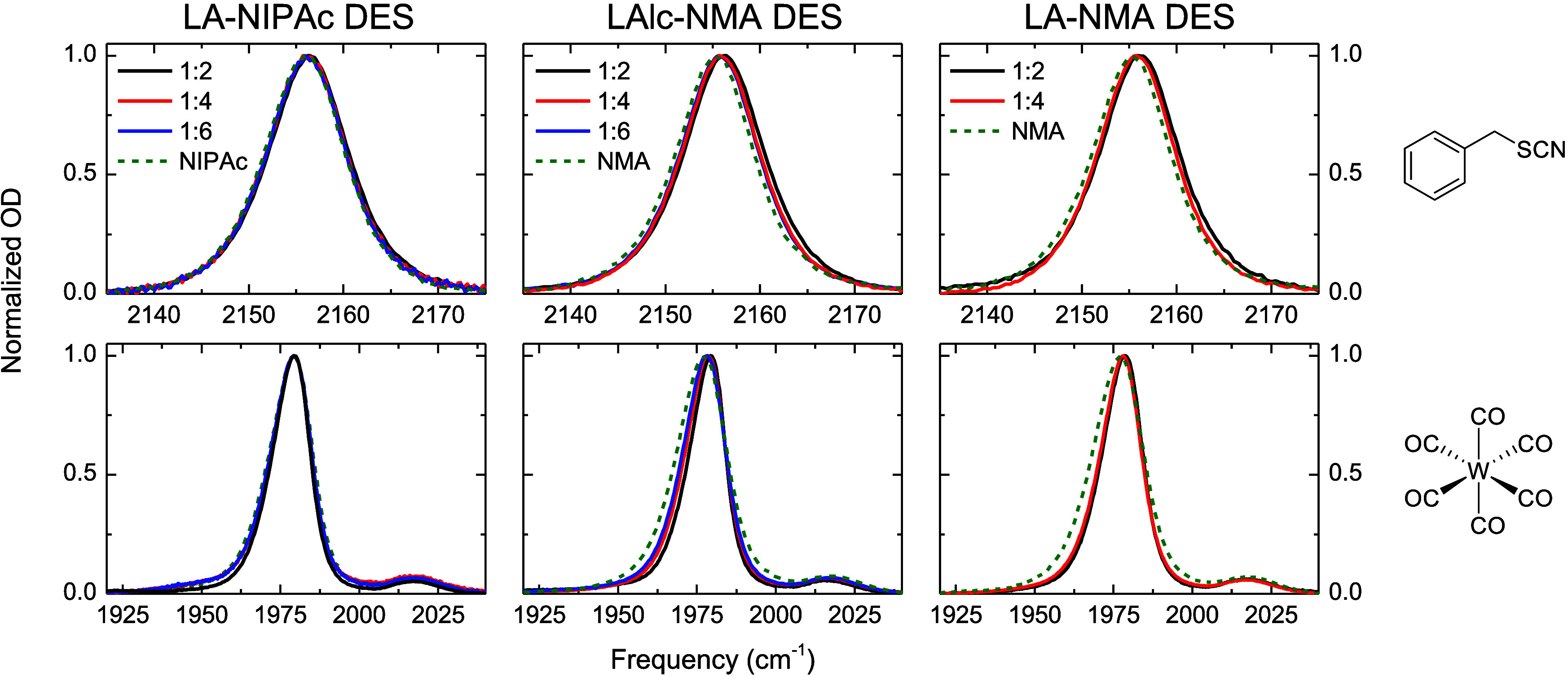
Normalized FTIR of the BSCN nitrile stretch (top row), and W­(CO)_6_ carbonyl stretch (bottom row) in LA-NIPAc (first column), LAlc-NMA (second column), and LA-NMA (third column). The last column is shown for comparison purposes only. Reproduced from ref 51 Copyright 2020 Americal Chemical Society. The IR spectra correspond to the 1:2 (black line), 1:4 (red line), and 1:6 (blue line) DES compositions, as well as to the pure amides (green lines). The molecular structures of BSCN, and W­(CO)_6_ are shown on the right.

The FTIR results demonstrate that both BSCN and W­(CO)_6_ remain solvated by NIPAc in LA-NIPAc mixtures regardless of the presence of LA in the sample. In contrast, the nonpolar and polar molecules relocate to the interface between the nonpolar (LAlc) and polar (NMA) domains in the LAlc-NMA DES, which is consistent with their behavior in the LA-NMA DES (Figure 3).^49–51^ Particularly, the slight blue shift (less than 2 cm^–1^, see Tables S1 and S2) for the peaks of the two solutes in the LAlc-NMA DESs with respect to that in pure NMA corresponds to these two molecules being located at the interphase of the LAlc and the NMA domains, as previously observed.^49–51^ In the LA-NIPAc DESs, the solutes remain in the polar domain, and the solubility of nonpolar solutes does not change with the formation of the nonpolar domain. These results support a mechanism of enhanced solubility produced by the presence of an organized interface between the crystalline-like domains of the nonpolar precursor and the amide in both the LA-NMA and LAlc-NMA DESs.

The generality of the enhanced solubility mechanism due to the solute location at an organized interface is tested by measuring the solubility of the nonpolar solute in a completely different DES system composed of LA and menthol at a molar ratio of 1:4,^60^ for which the LA-ME DES has been previously shown to present mesoscale heterogeneities in its molecular structure.^60^ Indeed, the solubility of W­(CO)_6_ increases by more than 20% from pure ME to the LA-ME DES (Figure 4). Moreover, analysis of the solvation environment of the nonpolar solute in the LA-ME DES via FTIR (Figure 4) reveals that the compound is in neither the ME nor the LA environment. Therefore, it is likely at the interface formed by the LA and ME in the mixture, as in the previously described cases of LA-NMA and LAlc-NMA DESs. Overall, these results show that creating an organized interface enhances the solubility of the nonpolar compounds in the LA-ME DES.

**Figure 4:**
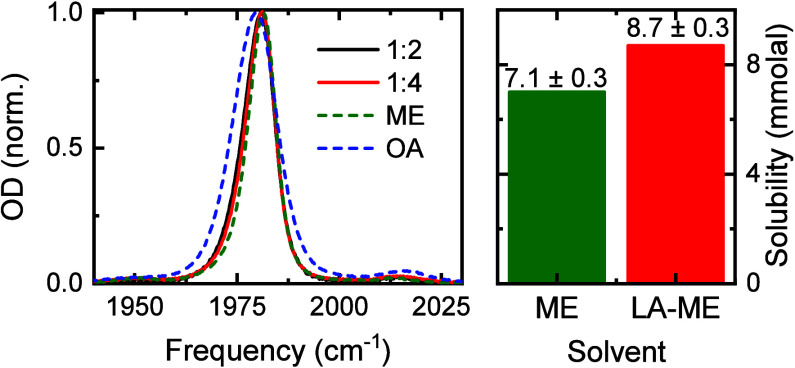
FTIR and solubility of the nonpolar compound in ME and ME-LA DES. Left panel shows the normalized FTIR of the W­(CO)_6_ carbonyl stretch in LA-ME in 1:2 (black), and 1:4 (red) molar ratios, pure ME (green), and octanoic acid (blue). Right panel shows the solubility of W­(CO)_6_ in pure ME (green) and in the LA-ME DES (red).

## Conclusions

This work shows that chemically modified LA-NMA DES precursors yield two new eutectic mixtures: LA-NIPAc and LAlc-NMA. However, only the latter and the unmodified DESs exhibit contrasting solubility of a nonpolar compound relative to its pure polar precursor. Microscopic and mesoscale characterizations of the DESs, as determined by FTIR and SAXS experiments, show the formation of microscopic heterogeneities due to the aggregation of the nonpolar DES component, although with significant differences. The heterogeneities in the LAlc-NMA DES appear to have the LAlc molecules arranged in an interdigitated manner similar to those in the LA-NMA DES. In contrast, the heterogeneities of the LA-NIPAc DES are smaller and more disorganized. Characterization of the solvation environment of two solutes with different polarities in the DESs supports similarities in the LAlc-NMA and the LA-NMA DESs, which differ from that of the LA-NIPAc DES. These differences arise from the solute's location, i.e., either interface or amide domain. It is therefore proposed that the enhanced solubility of the nonpolar solute in the different DESs is caused by the presence of an organized nonpolar–polar interface. Support for this proposed hypothesis was obtained by testing the solubility of the nonpolar solute in another molecularly heterogeneous DES, composed of LA and ME. All DESs with an organized structure show an increased solubility compared to the pure precursor. Overall, this study shows that the presence of ordered molecular heterogeneities and their corresponding interfaces in DESs enhances the solubility of nonpolar compounds. This opens the possibility of creating new media for separating substrates with varying polarity. Hence, this study provides a new degree of freedom that can be used to tune the DES solubility properties, a concept that has not been previously considered by the DES community. In this case, the solubility enhancement is achieved by making simple chemical changes to the precursors that directly alter the DES nanoscale organization.

## Supplementary Material



## Notes

Details on preparation and results of the calibration curve for the determination of the W­(CO)_6_ solubility, FTIR parameters of the BSCN and W­(CO)_6_ solutes in the studied DESs, change of fwhm of the W­(CO)_6_ FTIR peak vs LA concentration in the LA-NIPAc and Lalc-NMA DESs, and change of the concentration of W­(CO)_6_ in the LA-NMA DESs upon increase of LA concentration (PDF)
