# Supplementary material for: Tuning the Solvation and Solubility Properties of Molecularly Heterogeneous Nonionic Deep Eutectic Solvents via Interface Organization
Source: Langmuir. 2026 Mar 20;42(13):9070–8. doi: 10.1021/acs.langmuir.5c05846 (PMC13063817; doi:10.1021/acs.langmuir.5c05846)
Supplement: Supplementary file 1 [file la5c05846_si_001.pdf]

Supporting information

Tuning the Solvation and Solubility Properties of Molecularly Heterogeneous Nonionic Deep Eutectic Solvents via Interface Organization

Laura X. Sepulveda-Montaño, Chaila N. Estrella, Amelia M. Skinner and Daniel G. Kuroda*

Department of Chemistry, Louisiana State University, Baton Rouge, Louisiana 70803, United States.

*Address correspondence to dkuroda@lsu.edu.

Number of pages: 5

Number of tables: 3

Number of figures: 6

Table of Contents

**Sample preparation**	**S2**
**Tables**	**S2**
Concentration of $W(CO)_6$ in the different DESs	S2
FTIR parameters of the BSCN probe in the LA-NIPAc and LAlc-NMA DESs	S2
FTIR parameters of the $W(CO)_6$ probe in the LA-NIPAc and LAlc-NMA DESs	S3
**Figures**	**S4**
Calibration curve of $W(CO)_6$	S4
DSC curves for different compositions of LAlc-NMA, and for LA-NIPAc DESs	S4
Solubility of $W(CO)_6$ in LA-NMA DESs with different molar ratios	S4
FTIR of the amide region of the LA-NIPAc, and LA-NMA DESs	S5
Temperature dependent FTIR for a 1:4 molar ratio mixtures of LA-NIPAc	S5
Change in the FWHM of the $W(CO)_6$ peak in LA-NIPAc, and LAlc-NMA DESs	S5

Sample preparation

The solubility of $W(CO)_6$ in the different solvents was made through a calibration curve for $W(CO)_6$ in 1,4-dioxane which was prepared by successive dilutions from a 30 mm stock to achieve concentrations of 30, 20, 10, and 5mm. Furthermore, the determination of the solubilities was done by obtaining the areas of the peaks corresponding to the asymmetric CO stretch of the molecule. The determination of the solubilities was done by obtaining the areas of the peaks corresponding to the asymmetric CO stretch of the molecule. Errors were estimated from the calibration curve.

Tables

Table S1.Concentration of $W(CO)_6$ in the different DESs studied in the manuscript and their corresponding solubility errors.

Solvent	concentration (mmolal)	error
NMA	3.43	0.23
NIPAc	7.23	0.26
LA-NMA	5.79	0.25
LAlc-NMA	5.40	0.24
LA-NIPAc	7.15	0.26
LA-NMA (1:6)	4.39	0.24
LA-NMA (1:4)	4.61	0.24
LA-NMA (1:2)	4.70	0.24
ME	7.03	0.26
LA-ME	8.67	0.27

Table S2. FTIR parameters of the BSCN probe in the LA-NIPAc and LAlc-NMA. Central frequency of the peaks ω_0, and full width at half maximum (FWHM).

Molar ratio	ω_0 LA-NIPAc cm^{-1} ±0.02	FWHM LA-NIPAc cm^{-1} ±0.1	ω_0 LAlc-NMA cm^{-1} ±0.01	FWHM LAlc-NMA cm^{-1} ±0.1
1:2	2156.38	10.0	2156.20	10.1
1:4	2156.19	9.8	2155.89	9.8
1:6	2156.16	10.1	2155.77	9.8
0:1 (pure amide)	2156.99	10.1	2155.31	9.7

Table S3.FTIR parameters of the $W(CO)_6$ probe in the LA-NIPAc and LAlc-NMA. Central frequency of the peaks ω0, and full width at half maximum (FWHM).

Molar ratio (amide:C12)	ω_0 LA-NIPAc cm^{-1} ±0.03	FWHM LA-NIPAc cm^{-1} ±0.1	ω_0 LAlc-NMA cm^{-1} ±0.1	FWHM LAlc-NMA cm^{-1} ±0.1
1:2	1979.09	14.2	1978.8	13.6
1:4	1979.02	15.1	1978.2	15.3
1:6	1979.01	15.4	1977.8	16.3
0:1 (pure amide)	1978.99	16.3	1976.8	19.1

Figures

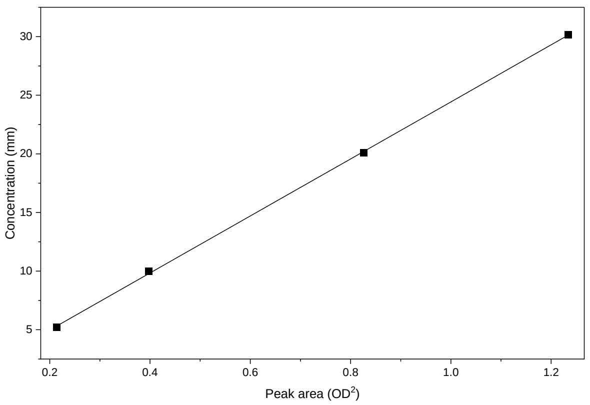

Figure S1. Calibration curve of $W(CO)_6$ in 1,4-Dioxane with equation y = (24.3±0.2)*x-(0.1±0.2), R=0.9998.

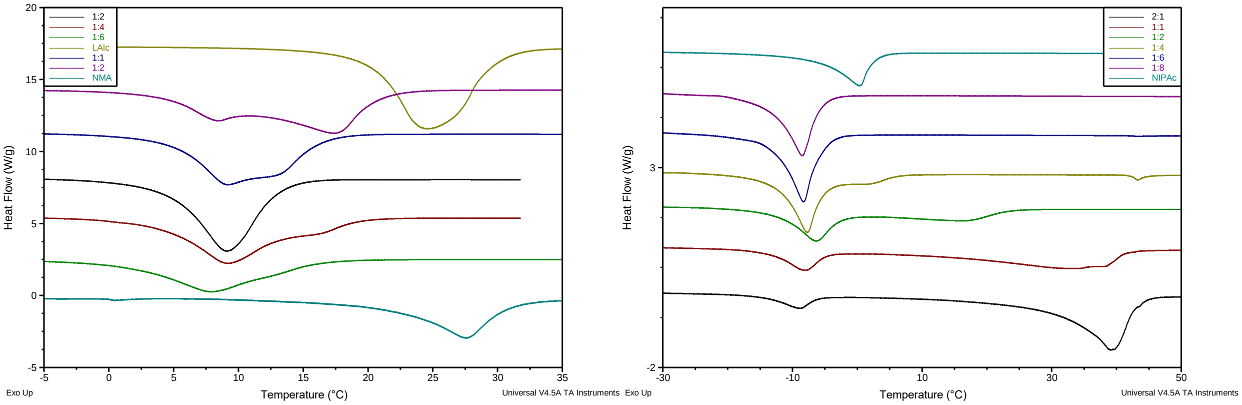

Figure S2. DSC curves for different compositions of LAlc-NMA (left), and for LA-NIPAc (right) used to build the phase diagrams presented in the main manuscript.

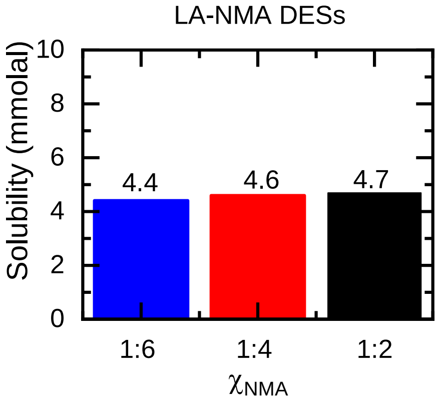

Figure S3. Solubility of $W(CO)_6$ in LA-NMA DESs with different molar ratios at room temperature.

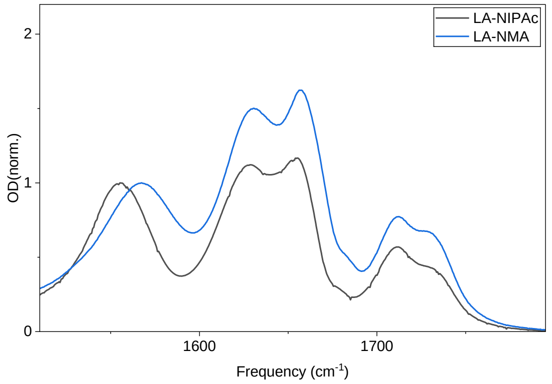

Figure S4. FTIR of the amide region of the LA-NIPAc (grey), and LA-NMA (blue) DESs in a 1:2 molar ratio, the spectra are normalized respect to the amide II mode of each sample.

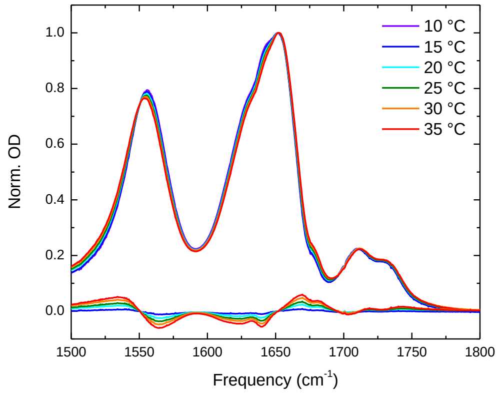

Figure S5. Temperature dependent FTIR for a 1:4 molar ratio mixtures of LA-NIPAc at temperatures of 10 (purple), 15 (blue), 20 (cyan), 25 (green), 30 (orange), and 35°C (red) in the amide I, II and carbonyl region.

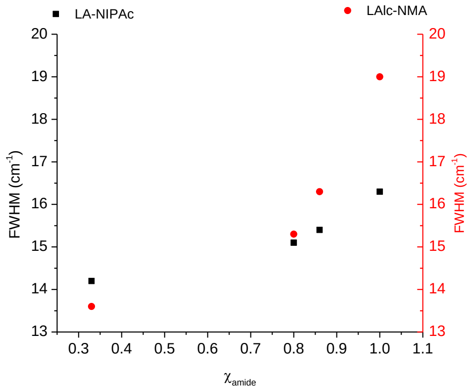

Figure S6. Change in the FWHM of the $W(CO)_6$ peak in LA-NIPAc (black squares, left axis), and LAlc-NMA (red circles, right axis) at different molar ratios.
